# Mercury Levels in Human Hair and Farmed Fish near Artisanal and Small-Scale Gold Mining Communities in the Madre de Dios River Basin, Peru

**DOI:** 10.3390/ijerph14030302

**Published:** 2017-03-14

**Authors:** Aubrey L. Langeland, Rebecca D. Hardin, Richard L. Neitzel

**Affiliations:** 1 School of Natural Resources and the Environment, University of Michigan, Ann Arbor, MI 48109, USA; aubreyll@umich.edu (A.L.L.); rdhardin@umich.edu (R.D.H.); 2 Department of Environmental Health Sciences, School of Public Health, University of Michigan, Ann Arbor, MI 48109, USA

**Keywords:** mercury, exposure assessment, human health, artisanal and small-scale gold mining

## Abstract

Artisanal and small-scale gold mining (ASGM) has been an important source of income for communities in the Madre de Dios River Basin in Peru for hundreds of years. However, in recent decades, the scale of ASGM activities in the region has increased dramatically, and exposures to a variety of occupational and environmental hazards related to ASGM, including mercury, are becoming more widespread. The aims of our study were to: (1) examine patterns in the total hair mercury level of human participants in several communities in the region and compare these results to the 2.2 µg/g total hair mercury level equivalent to the World Health Organization (WHO) Expert Committee of Food Additives (JECFA)’s Provisional Tolerable Weekly Intake (PTWI); and (2), to measure the mercury levels of paco (*Piaractus brachypomus*) fish raised in local aquaculture ponds, in order to compare these levels to the EPA Fish Tissue Residue Criterion of 0.3 µg Hg/g fish (wet weight). We collected hair samples from 80 participants in four communities (one control and three where ASGM activities occurred) in the region, and collected 111 samples from fish raised in 24 local aquaculture farms. We then analyzed the samples for total mercury. Total mercury levels in hair were statistically significantly higher in the mining communities than in the control community, and increased with increasing geodesic distance from the Madre de Dios headwaters, did not differ by sex, and frequently exceeded the reference level. Regression analyses indicated that higher hair mercury levels were associated with residence in ASGM communities. The analysis of paco fish samples found no samples that exceeded the EPA tissue residue criterion. Collectively, these results align with other recent studies showing that ASGM activities are associated with elevated human mercury exposure. The fish farmed through the relatively new process of aquaculture in ASGM areas appeared to have little potential to contribute to human mercury exposure. More research is needed on human health risks associated with ASGM to discern occupational, residential, and nutritional exposure, especially through tracking temporal changes in mercury levels as fish ponds age, and assessing levels in different farmed fish species. Additionally, research is needed to definitively determine that elevated mercury levels in humans and fish result from the elemental mercury from mining, rather than from a different source, such as the mercury released from soil erosion during deforestation events from mining or other activities.

## 1. Introduction

### 1.1. Overview of Artisanal and Small-Scale Gold Mining

Artisanal and small-scale gold mining (ASGM) is a term broadly used to describe the gold mining by individuals, families, or groups with minimal mechanization, often in the informal or illegal sector of the market [1]. ASGM occurs in over 70 developing countries [2]. It is estimated to employ 13 million people globally, and an additional 80–100 million people are directly reliant upon or impacted by ASGM [3].

In the Peruvian Amazon, where gold deposits are typically alluvial in nature, ASGM has been a source of income for local populations for centuries [4]. However, the Peruvian region of Madre de Dios has seen a drastic increase in ASGM, and a commensurate increase in the population, over the last three decades [4]. In 2011, there were estimated to be more than 80,000 ASGM workers in Peru, plus an additional 300,000 workers employed in peripheral services [5]. For example, the population of the region is estimated to have roughly doubled every 20 years from 1940 to 2015 [6]. Madre de Dios produced close to 70% of Peru’s artisanal gold in 2001 [7]. However, in 2011, an estimated 97% of mining concessions in the region were illegal (in [8]). The environmental impacts of these illegal ASGM activities are substantial, and include deforestation [9,10], habitat loss and desertification [11], and air and water pollution from gasoline and oil spills and combustion [12]. The region is also touted as one of the world’s greatest biodiversity hotspots [13], further increasing the impacts of the environmental degradation associated with ASGM.

### 1.2. ASGM and Mercury

ASGM activities involve the extensive use, and subsequent environmental release, of inorganic elemental mercury, which is used to amalgamate fine alluvial gold particles and increase gold recovery. Elemental mercury is burned off of the gold amalgam before sale, to ensure a pure gold product [14]. Although some larger operations use retorts or fume hood condensers to capture and recycle mercury vapors, it is common for ASGM workers to burn mercury indoors, often in homes, without ventilation, which can result in substantial inhalation exposure [14]. After elemental mercury is vaporized, it can enter into aquatic ecosystems, where it may be biomethylated by bacteria into an organic form, methylmercury [15]. Fish and macroinvertebrates in aquatic ecosystems accumulate methylmercury in their tissues, with increasing concentrations in higher trophic levels. At least 95% of the total mercury present in fish tissue is methylmercury [16]. Elemental mercury can also directly enter aquatic ecosystems, without undergoing vaporization; an estimated 45% of the mercury used in informal and illegal ASGM activities in the Amazon region of Brazil is directly lost as a result of spills or dumping [17]. Human populations in Madre de Dios depend upon fish as a dietary staple, and fish consumption is the main route of exposure to methylmercury [18].

Global estimates cite ASGM as contributing approximately 17% of anthropogenic mercury emissions, and it is responsible for further mercury contamination through direct spillage into the environment [2,19]. In 2010, ASGM was the major source of global mercury emissions to air, releasing 727 tons per year [20]. Peruvian mercury imports exponentially increased from 2003 to 2009 [9], and from 2006 to 2009 it was estimated that 95% of this imported mercury was used in ASGM (in [7]). In October 2013, Peru signed the United Nations’ Minamata Convention, an international treaty to reduce anthropogenic mercury emissions [21]. However, Peru’s mercury imports have increased in recent years, largely for use in ASGM [9]. A distinction is made in Peru between informal and illegal mining. Informal mining is used to describe mining operations that take place in legally-designated mining zones, but without a formal government-issued permit. Illegal mining—that occurring outside of legally-designated mining zones—makes up the majority of mining in the Madre de Dios region [22]. The Peruvian government has been unable to control the spread of illegal mining over the last decade. A reported 450 hectares have been deforested within the Tambopata National Reserve as of September 2016 [23]. In May 2016, the government of Peru issued a 60-day public health emergency after studies showed that up to 48,000 people across more than 85,000 hectares are affected by mercury exposure due to mining [24].

Mercury exposure is associated with a wide range of adverse human health effects, including neurological, cardiac, motor, reproductive, genetic, renal, and immunological conditions [25,26,27,28,29,30,31]. Exposure to elemental mercury vapors can result in tremors, a decrease in memory performance, and a decrease in autonomic nervous system functioning [32]. Exposure to methylmercury can cause damage to the central nervous system [25,33]. Developing fetuses and young children are at a particularly high risk of adverse neurological effects from methylmercury [34], and maternal methylmercury can be transferred to a fetus in the womb [35].

Human mercury exposure may be assessed through assays of urine (useful for evaluating elemental mercury exposure) and blood (useful for measuring methylmercury exposures from dietary sources and elemental exposures) [36,37]. The total mercury in human hair is a good indicator of long-term mercury exposure, particularly for methylmercury [38,39]. The concentration of hair methylmercury is proportional to the blood concentration at the time of the formation of the hair strand [40], with the concentration measured in hair being approximately 250 times that of the corresponding blood level. The Joint Food and Agriculture Organization of the United Nations (FAO) and the World Health Organization’s (WHO) Expert Committee of Food Additives (JECFA) set a Provisional Tolerable Weekly Intake (PTWI) of 1.6 µg methylmercury per kg of body weight. The PTWI was set at a level considered sufficient to protect developing fetuses, the subgroup most vulnerable to the effects of mercury exposure [41]. The PTWI is associated with a total hair mercury concentration of approximately 2.2 µg mercury/g dry hair; we used this level as a comparison for our adult populations [42].

### 1.3. ASGM in Madre de Dios

Several previous studies have evaluated human exposure to mercury in Madre de Dios. While ASGM comprises a significant source of mercury in the environment in this region, there are also non-anthropogenic sources and geochemical effects to consider. For example, hair mercury levels have been shown to be positively correlated with river pH and dissolved organic carbon in the Amazon basin [43]. Elevated mercury levels in the Madeira River (into which the Madre de Dios River flows, via the Beni River) are largely due to natural sources and biogeochemical processes [44]. However, for the purposes of this study we will review the literature that has investigated mercury in the region as related to ASGM activities.

One unpublished study demonstrated that the average hair mercury levels exceeded the U.S. EPA Reference Concentration for human hair of 1 µg/g [45], and a second study showed that hair mercury levels were higher in ASGM areas than in non-mining areas [46]. High levels of mercury have also been found in the blood and urine of ASGM miners in Madre de Dios [47]. Additionally, hair mercury levels have been associated with a higher fish consumption [46,47]. The U.S. EPA set a methylmercury Tissue Residue Criterion for fish tissue intended for human consumption at 0.3 µg MeHg/g fish tissue (wet weight) [48]. The EPA estimates that about 95% of the total mercury present in fish tissue is methylmercury, yielding a reference dose for total mercury in fish tissue of 0.333–0.4 µg Hg/g fish tissue (wet weight) [48]. Studies of mercury and methylmercury in fish from rivers in Madre de Dios have shown ambiguous results, with some indicating levels below the EPA criterion [49], and some indicating elevated exposures [45,50,51], depending on the fish species sampled and the location of capture. A relatively recent development in Madre de Dios is the introduction of pond aquaculture practices [52] to improve natural fluvial stocks [53] and provide income alternatives to ASGM [54]. One of the most commonly farmed and consumed fish species in Madre de Dios is a native species known locally as “paco” (*Piaractus brachypomus*), favored for its performance in fish farms [55]. Mercury biomagnifies in aquatic food webs, as methyl mercury is retained by lower trophic levels and passed on to higher trophic levels after predation or consumption [56]. Paco is an omnivorous fish and, assuming that the fish raised in ponds on pelleted diets mimic omnivorous diets, we did not expect to find high mercury concentrations under natural environmental conditions.

Our study examined the impacts of ASGM in the Madre de Dios river basin of Peru, and appears to be the first to examine the relationship of the distance of various communities from the river headwaters, as well as to assess male and female exposure, without a particular occupational focus. The first aim of our study was to identify the risk factors for elevated total hair mercury levels of human participants in several communities along the length of the Madre de Dios River or located on tributaries, and to compare the observed levels of mercury in hair to the total hair mercury concentration of 2.2 µg/g, corresponding to the PTWI. We hypothesized that hair mercury levels would increase with increasing geodesic distance from the headwaters of the river. We used the geodesic distance, even for communities located on tributaries, to see if there was a trend of mercury concentration in human populations as one moved farther away from the headwaters, where little or no mercury contamination from ASGM should be present. Our second aim was to measure the mercury levels of the paco raised in local fish ponds in Madre de Dios, and to compare these levels to the EPA Fish Tissue Residue Criterion of 0.3 µg Hg/g fish (wet weight), which is the concentration of methylmercury in fish tissue that can be consumed without the expectation of adverse health outcomes (based on the assumption of 0.0175 kg fish/day) [48]. We hypothesized that the fish in the pisciculture ponds would have a mercury concentration above the tissue residue criterion.

## 2. Materials and Methods 

**Figure 1:**
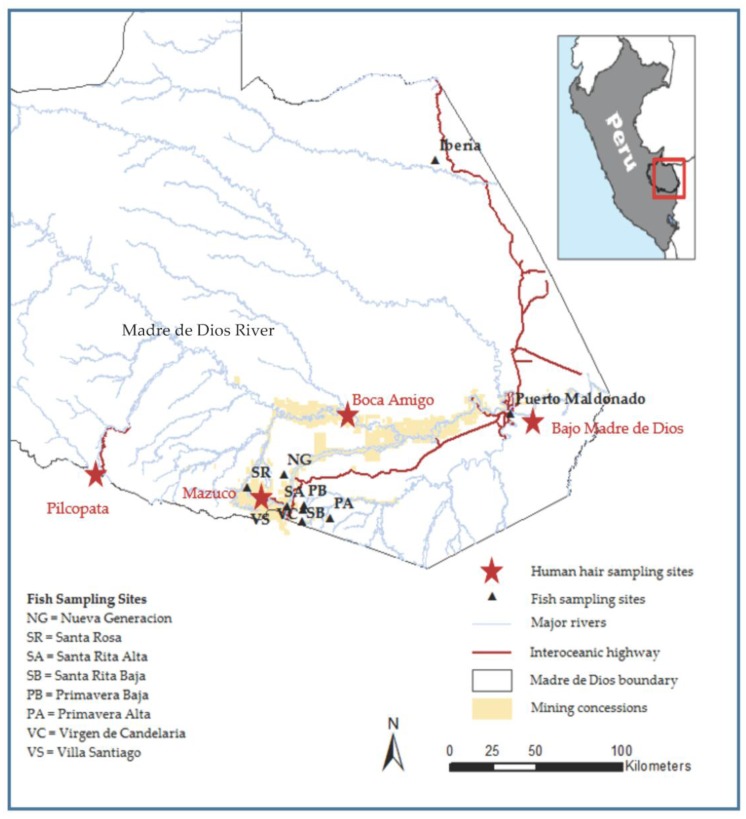
Map of the study site locations in the Madre de Dios river basin of Southeastern Peru. Map created with ESRI ARCGIS software, with layers downloaded from Atrim Biodiversity Information Systems [58].

Data were collected from May to July 2014 in four Peruvian communities along the 1060 km long Madre de Dios River, which originates in Peru [57] and empties into the Beni River in Bolivia (Figure 1). All research protocols involving human subjects were approved by the University of Michigan IRB (HUM00086592). All subjects gave their informed consent for inclusion before participating in the study.

### 2.1. Site Selection and Participant Recruitment

Field sites for human subjects were chosen to represent a range of distances from the headwaters of the Madre de Dios River (Table 1). The four sites were Bajo Madre de Dios, Boca Amigo, Mazuco, and Pilcopata. Pilcopata, the community closest to the headwaters, was chosen as a control site, due to the lack of nearby ASGM activities; as determined through an evaluation of satellite images from 2005 to 2014. Landsat data are distributed by the Land Processes Distributed Active Archive Center (LP DAAC), located at USGS/EROS, Sioux Falls, SD, USA. The presence of illegal mining in the other three communities was determined through an evaluation of satellite images and through conversations with community members. Formal demographic and population data were not available for all of the communities at the time of the study. The communities were generally similar, with the exception of proximities to mining and the degree of urbanization; Boca Amigo and Bajo Madre de Dios were not urban. Mazuco was the largest community sampled.

In the small communities of Pilcopata, Bajo Madre de Dios, and Boca Amigo, households near the main town center were visited for recruitment via convenience sampling. In the larger town of Mazuco, a section of the community was selected based on convenient access for researchers and all houses in that section were approached for recruitment. At all four sites, one (and, in rare cases, two) adult participated in each sampled household, with a range of 12 to 19 households visited per site. Our a priori sampling goal was 20 subjects from 20 households, in each of the four communities.

### 2.2. Human Subjects Survey

The survey completed by all subjects addressed demographic factors (age, sex, study site, education, duration of residence, household size, pregnancy, etc.); the frequency (number of meals per week including fish) and source of different types of fish consumed; the number of servings of fish consumed in the three days prior to the survey; and the frequency and source of other types of protein consumed. Note that, due to the often-illegal nature of mining in the area, we chose not to ask participants about their mining activities. The study inclusion criteria were an age of at least 18 years and residence in the study site for at least six months.

### 2.3. Human Hair Sample Collection and Analysis

After completing the survey, a collection of roughly 200 strands of hair (the approximate number needed to obtain the necessary mass of hair for subsequent analysis) were cut from the occipital region of the skull of each participant. Samples were taken with stainless steel scissors as close to the scalp as was safely possible. The hair was stored between sheets of adhesive paper, and the sample end closest to the scalp was marked. The samples were labeled, stored individually in double plastic Ziploc bags, and frozen after transport to the University of Michigan and prior to analysis.

Human hair samples were analyzed at the University of Michigan using a Milestone Direct Mercury Analyzer (DMA-80, Milestone Inc., Shelton, CT, USA), using EPA method 7473 [59]. Samples were trimmed so that the 4 cm length of hair closest to the scalp would be analyzed. Hair grows at an average rate of one cm per month [60], so our analysis was intended to estimate the total mercury exposure for the four-month period preceding analysis. A 50–55 mg hair sample was measured and placed in the DMA for analysis. Three different readings were taken for each sample, and the three runs were averaged to estimate the total mercury concentration per sample. Quality control (QC) measures included the random testing of reference materials (IAEA-086), blank measurements every 10th reading, and random checks of previously measured samples. Recovery rates of 95%–100% were considered acceptable for reference material tests. The Limit of Detection (LOD) was 0.003 ng Hg.

### 2.4. Fish Sample Site Selection and Sample Collection

Fish samples were collected from aquaculture farms throughout the Madre de Dios region (Table 2). Farms were selected based on convenience and awareness of their existence by local governmental and non-governmental agencies. We purchased recently-caught and killed fish that were to be consumed by the farm owner, directly from fish farmers. Fish from farms were targeted because heavy rainfall during the study period prevented fisherman from obtaining fish from the rivers. The selection of fish farm locations was not consistent with the sites where the human participants were sampled, due to constraints in access to refrigeration. The Iberia site, farthest from the Madre de Dios River and near the Brazilian border, served as a reference site, because it was believed to be similar to other study sites, but not subject to ASGM mercury. At the time of the study, no large-scale ASGM activities were believed to occur in Iberia. This study only focused on the fish species “paco”, as it is easy to find in fish farms throughout the region, allowing for a spatial comparison within one fish species.

Five paco specimens were purchased from pisciculture farmers on the same day as the fish had been caught and killed for their own consumption. All five samples were collected from a single pond from each fish farm, using standard fishing practices. Fish are purchased from NGOs and governmental agencies as fingerlings by the farmer. All fish are added to ponds at the same time, and are harvested in nets one year later. Therefore, farmers were able to tell us exactly how long fish had been in their respective ponds. The information gathered for each fish sample included the number of months in the pond, length (cm), approximate weight (kg), and GPS coordinates of the pond. A tissue sample weighing approximately 50 g was taken from the side of the deceased fish, over the lateral line just anterior to the tail. The sample was placed in a Ziploc bag and labeled; the remainder of the fish was either purchased or returned to the pond owners. All of the fish samples were frozen locally and then shipped to the University of Michigan on dry ice.

### 2.5. Fish Sample Laboratory Analysis

At the University of Michigan, fish samples were measured on a balance (to ±0.0001 g) while still frozen, then placed into individual Whirl Pak bags, labeled, and dehydrated for at least 48 h using a vacuum freeze dryer dehydrator. The final dry weights (to ±0.0001 g) were recorded after dehydration; these weights were, on average, 22%–25% of the wet weight. The dried samples were then pulverized to a fine powder and sampled using a disposable spatula. Samples were analyzed using the DMA-80 and EPA method 7473 [59]. As with the human hair samples, three different readings were taken and averaged for each fish sample. QC was similar to that used for human hair; for reference materials (IAEA-407, IAEA-436), recoveries between 90% and 110% were considered acceptable. The LOD was again 0.003 ng Hg.

The mercury concentration in fish tissue was converted from the dry weight concentration (i.e., the DMA output) to the wet weight concentration using Equation (1).

The conversion of dry weight [Hg] to wet weight [Hg] was as follows:

Wet weight [Hg] = (Dry weight [Hg] × (1 − Δweight))/100,
(1)

where Δweight is the change in weight for each sample between dry and wet weight measurements.

### 2.6. Statistical Analysis

Statistical analysis was performed using RStudio (RStudio, Boston, MA, USA) and Stata 14.1 (StataCorp LLC, College Station, TX, USA). Descriptive statistics were computed for all variables, and univariate and bivariate relationships were examined visually, through the use of histograms, scatterplots, and quantile plots, in combination with correlation coefficients. Non-normally-distributed variables were log-transformed prior to parametric statistical analyses; for these variables, the geometric mean and geometric standard deviation (GSD) are reported, in addition to the arithmetic mean and standard deviation. For inferential statistical tests, results were considered significant when *p* < 0.05.

To analyze the measured total mercury in human hair, ANOVA tests were used to test differences in mercury levels by categorical variables, using both the original response scales (two to four possible response categories, depending on the survey item) and the results after collapsing them into binary categories. ANOVA tests were also run on categorical variables and mercury levels after log-transforming the mercury levels. Chi-squared tests were used to test differences in the fraction of hair mercury measurements exceeding the 2.2 μg/g PTWI total hair mercury equivalent level by site and sex. Linear regression analyses were used to evaluate the bivariate association between the measured human hair total log-transformed mercury levels and each of the independent variables (i.e., demographic factors, community site, fish consumption, etc.), and logistic regression analyses were used to evaluate the odds ratio (OR) for total hair mercury levels >2.2 µg/g associated with each of the individual independent variables. A forward stepwise regression approach was used to develop multivariate linear and logistic regression models; variables were retained where *p*-values of 0.10 or less were observed. To account for the few cases where multiple adults were sampled in a single household, regression models accounted for the intragroup correlation of data by household (Stata “cluster” option).

For the fish samples, the total mercury (Hg mg/kg dry weight) was plotted against the fish specimen weight mercury, and the R-squared value and slope of the relationship were assessed. Descriptive statistics were computed for the mean wet weight mercury concentration (mg/kg), both overall and by study site. The fraction of samples exceeding the EPA tissue residue criterion of 0.3 µg MeHg/g fish tissue was computed, both overall and by sampling site.

## 3. Results

### 3.1. Human Survey Results

Study participation rates were not formally assessed. The most common reason given for non-participation was a lack of interest in providing a hair sample, either due to aesthetic considerations or cultural beliefs. It is possible that some individuals may have declined to participate because they were engaging in mining themselves; however, this was never stated as a reason to the researchers. Table 3 shows information about the 81 participants, both overall and by site. Of the 81 adult participants, 12 (14.8%) were sampled from six households; all other participants were the only adults sampled in their households. Just under half of the participants (39%, or 48.2%) were male. Participants were roughly equally distributed across the four sites. The mean age, mean residence time, and total household size differed among the four sites, but were not statistically significant. The number of children per household significantly differed among sites, with Pilcopata having the highest value, and Boca Amigo the lowest value. Education levels differed by site: Pilcopata had by far the largest fraction of participants that had completed more than secondary school (data not shown). Mazuco and Pilcopata had the largest fraction of participants that had completed secondary school, and Bajo Madre de Dios had by far the largest fraction of participants that had not completed primary school.

The frequency of fish consumption was correlated with the number of servings of fish in the three days prior to the survey (Spearman correlation coefficient, 0.41, *p* = 0.002), suggesting that the number of meals containing fish in the prior three days was a useful surrogate measure of fish consumption. Fish and chicken consumption were not correlated (spearman correlation coefficient, −0.12, *p* = 0.89). Mean servings of fish in the past three days did significantly differ among sites, with Boca Amigo having the highest mean value and Pilcopata the lowest. Finally, the consumption of different fish species (reported in free-text responses to the questionnaire) indicated that the fish species paco and bagre did not differ significantly among sites, but that the consumption of boca chico, sabalo, doncella, and zungaro did. Participants in Pilcopata were the least likely to consume any of these four types of fish; participants in Mazuco were the most likely to consume boca chico and sabalo, while participants in Boca Amigo were the most likely to consume doncella and zungaro.

**Figure 2:**
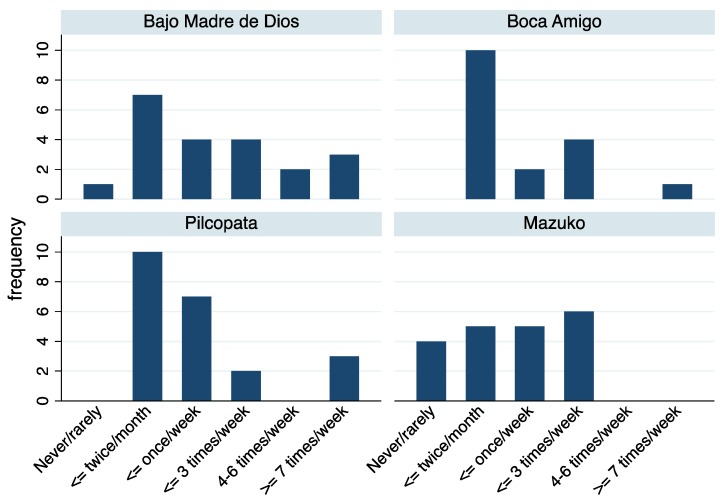
Percent of participants reporting categories of fish consumption frequency at four study sites in the Madre de Dios river basin of Southeastern Peru.

Figure 2 shows the distribution of the reported frequency of fish consumption by study site. Boca Amigo and Pilcopata had the greatest fraction of participants reporting an infrequent (i.e., two times per month or less) consumption of fish, while Bajo Madre de Dios and Pilcopata had the greatest fraction of participants reporting very frequent consumption (i.e., greater than or equal to seven times per week).

### 3.2. Human Hair Mercury Results

Table 4 shows the descriptive statistics for the total mercury levels in human hair. All of the collected hair samples had a sufficient mass for laboratory analysis. The control site, Pilcopata, had the lowest arithmetic and geometric mean and standard deviation, and there was only a single individual at Pilcopata that had mercury readings above the PTWI total hair mercury equivalent level of 2.2 µg/g. The highest arithmetic and geometric mean total mercury levels were found at the downriver mining sites Boca Amigo and Bajo Madre de Dios; these two sites also had the highest measured values of any of the four sites. At both Boca Amigo and Bajo Madre de Dios, two-thirds or more of the hair samples exceeded the reference level. ANOVA results indicated that the arithmetic and geometric mean levels of total hair mercury differed among the four sites. No significant differences in arithmetic or geometric mean levels of total hair mercury were noted when considering sex, either overall or within any of the four sites. The fraction of samples exceeding the reference level significantly differed among sites. No significant differences in the fraction of samples exceeding the EPA limit for total hair mercury were noted between sexes, either overall or within a site.

Unadjusted linear regression models using a single dependent variable regressed on the log-transformed total hair mercury level, yielded *p*-values > 0.05 for nearly all variables. However, the coefficients for three of the four sites (reference site: Pilcopata) yielded *p*-values < 0.0001. Each of these site indicator variables showed positive coefficients for log-transformed total hair mercury levels, when compared to the reference site. These variables were selected for inclusion in an adjusted linear regression model.

A similar approach was used for logistic regression, with the dependent variable being the total mercury hair level in excess of the 2.2 µg/g PTWI total hair mercury equivalent level. The majority of independent variables assessed did not reach statistical significance. However, as with the linear regression models, the coefficients for three of the four sites (reference site: Pilcopata) reached statistical significance. These variables were selected for inclusion in an adjusted logistic regression model.

Table 5 shows the results of the multivariable adjusted linear and logistic regression models. Linear regression model one included the only variable (site) that reached statistical significance. The two farthest downriver ASGM communities (Bajo Madre de Dios and Boca Amigo) both had substantially larger coefficients than the upriver community (Mazuco), and all three of these communities had elevated levels of mercury compared to the reference community (Pilcopata). Adjustment for other factors, including age, sex, education level, frequency and type of fish consumption, and frequency and type of other protein consumption, only resulted in marginal improvements to the model. Model two is adjusted for age and sex, and had an essentially identical model fit (0.51 R^2^).

Logistic regression model three shows the only variable (site) that reached significance in the logistic regression analysis. Consistent with the results of linear regression models one and two, logistic regression model three showed that the two farthest downriver ASGM communities (Bajo Madre de Dios and Boca Amigo) both had substantially larger ORs than the upriver community (Mazuco), and all three of these communities had elevated levels of mercury compared to the reference community (Pilcopata). Participants in Boca Amigo were 5.9 times more likely to have mercury levels in excess of the 2.2 µg/g reference level than those in Pilcopata. As with the linear regression models, adjustment for other factors (e.g., age, sex, education level, frequency and type of fish consumption, and frequency and type of other protein consumption) resulted in negligible improvements to the model. Logistic regression model four, which adjusted for age and sex, achieved a virtually identical pseudo-R^2^ (0.40).

### 3.3. Fish Sample Results

**Figure 3:**
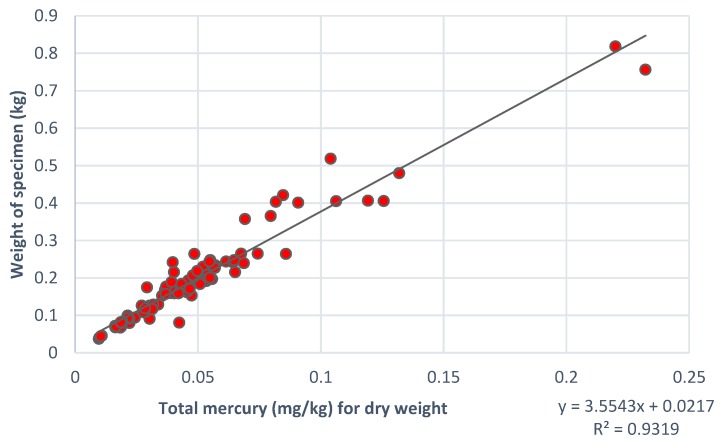
Scatterplot showing the association between total mercury concentration (dry weight) and total fish weight for all fish tissue samples, at 24 sampling sites in the Madre de Dios river basin of Southeastern Peru.

The weight of the fish specimen was positively correlated with mercury (mg Hg/kg dry fish tissue) (Figure 3). The R^2^ value of the relationship between fish specimen weight in kg and total mercury concentration in dry fish tissue, was 0.932. The fish sampled were between approximately four and eight months old at the time of sacrifice (i.e., fingerling transplant to aquaculture pond plus four to eight months of growth).

The arithmetic and geometric mean total mercury concentration for all 111 samples across the four study sites are shown in Table 6. All samples were below the EPA Tissue Residue Criterion of 0.3 mg/kg. Of all the samples, only two fish from Virgen de la Candelaria had mercury concentrations near this value (0.23 and 0.22 mg Hg/kg wet weight fish tissue). As shown in Table 6, Mazuco had the lowest mean mercury concentration for both wet weight and dry weight (0.028 mg/kg and 0.106 mg/kg, respectively). The site with the highest mercury concentration was Virgen de la Candelaria (Ww = 0.12 mg/kg Hg and Dw = 0.40 mg/kg Hg). The control site of Ibería had a mean wet weight of 0.04, making it the third lowest concentration. Figure S1 in the supplementary materials provides a visual representation of these data.

Finally, when mercury levels were modeled by approximate age of fish, the average mercury levels were seen to increase by approximately 0.01 mg/kg per year (data not shown). Fish of approximately four months of age had mean levels of 0.04 mg/kg wet weight, increasing to 0.06 mg/kg at six months of age, and 0.08 mg/kg at eight months of age. Thus, it appears that, as the fish increased in size and had increasing residence time in aquaculture ponds, their mercury levels increased in a roughly linear manner. If this linear trend were to continue through one year of age (the typical age at harvest), the total mercury content would be approximately 0.12 mg/kg, which is still well below the EPA tissue residue criterion of 0.3 mg/kg.

## 4. Discussion

The results of this study suggest that people living in communities in the Madre de Dios region of Peru where ASGM activities occur are subjected to higher mercury exposure than those living in a non-mining community. Residence in a ASGM community was associated with highly increased odds of total hair mercury levels exceeding the reference level of 2.2 µg Hg/g hair (ORs of 5.8, 3.8, and 2.2 for participants in Bajo Madre de Dios, Boca Amigo, and Mazuco, respectively). These ORs suggest a dose-response relationship, with the communities furthest from the headwaters of the Madre de Dios River (Bajo Madre de Dios and Boca Amigo) having the highest odds of exceeding the 2.2 µg/g PTWI total hair mercury equivalent level. Measurements of paco purchased from aquaculture farms in the region did not identify any samples with levels of mercury that exceeded the EPA tissue residue criterion of 0.3 μg Hg/kg wet weight. The site used as a control, Iberia, did not have the lowest levels of mercury in sampled fish, but levels from this site were among the lowest of all sites evaluated. Collectively, these results suggest that ASGM activities are associated with higher human mercury exposure, and that farmed fish contribute little to mercury exposure.

Our human hair analysis results are generally consistent with several prior studies that have evaluated human exposure to mercury in Peru. Among 226 adults from the capital city of Madre de Dios, Puerto Maldonado, the average hair mercury concentration was two to three times higher than the reference level of 2.2 µg Hg/g hair. [45]. A 2012 study on the mercury levels in human hair among 104 participants in the town of Puerto Maldonado in Madre de Dios and 100 participants in an ASGM mining zone in the region, found that residence location and sex were correlated with higher mercury levels, and that the total levels of mercury in hair were significantly higher in the mining zones [46]. A study of 103 ASGM miners in Madre de Dios in 2010 found that all participants had detectable levels of mercury in urine, and 91% had detectable levels of blood methylmercury [47]. As with the study by Ashe (2012) [46], Yard et al. (2012) [47] found that higher fish consumption was associated with increased hair mercury levels; Yard et al. further found that exposure to heated gold-mercury amalgam was correlated with higher hair mercury levels.

Our results do not match those of similar studies in concluding that the amount of fish consumed correlates with increased mercury levels. It is possible that, due to the timing of this study, fish was not currently in season and had not been for several months. Therefore, the level of total mercury may be higher in the study populations during the dry season, when fishing is more lucrative and diets depend more heavily upon river fish as a staple food. Additionally, the metric for fish consumption used in this study relied on self-reported dietary behaviors, and may be susceptible to recall or other reporting biases. Other studies have also shown a correlation between sex and total hair mercury levels [45,46]; we did not identify such a relationship here.

The fish sample results were consistent with two of four previous studies of mercury concentrations in fish in the Madre de Dios region. A 1997 study measured the level of mercury and methylmercury in samples from seven fish families in the Manu River in Madre de Dios. The total mercury levels in two samples of paco were 0.053 and 0.067 µg Hg/gram wet weight [49]—i.e., below the EPA tissue residue criterion, and similar to those found here. The total mercury concentrations in paco purchased from markets in the capital of Madre de Dios, Puerto Maldonado, were below the EPA criteria for methylmercury (mean 0.24 mg/kg) [45], though it is important to note that fish sold at this market may not have been locally caught or raised. Two other studies of species of fish which are common in the Madre de Dios river found average total and methylmercury concentrations above the EPA tissue residue criterion [45,51]. Future research needs to include an investigation on different fish species, as well as different fish sources (farmed versus river caught, etc.). In addition, future research should consider the characteristics of individual ponds, such as pond age, source of water used, distance of pond from mining activities, fish fry source, etc., to eliminate possible confounding.

Our study has several important limitations. First, the illegal and informal nature of mining and political circumstances may have influenced participation rates among households in the four research sites. If so, this would have negatively biased the total hair mercury results presented here, as households actively engaged in mining may have been less likely to participate. Our methods of recruitment meant that we were not able to compute quantitative participation rates. Second, our study utilized convenience sampling instead of random sampling. This limits the generalizability of our findings. Third, the fish samples analyzed were well below the one-year age at which aquaculture fish are typically harvested for market sale, which may have negatively biased their measured mercury levels. Fourth, there was a potential temporal mismatch between our survey items related to fish and other protein consumption, which were intended to assess consumption in a typical week, and the measurements of hair mercury, which reflected mercury exposure over the several months prior to sample collection. Finally, our regression model development approach used a conventional statistical approach (i.e., binary cutoff *p*-values for stepwise model building). This approach may have resulted in some potentially important risk factors being discarded during our model building efforts [61,62], but was considered appropriate given our small sample size and the exploratory nature of our analyses. Nevertheless, our finding that the total hair mercury levels were statistically significantly higher in communities where ASGM activities occur, supports the idea that anthropogenic activities increase the risk of exposure to mercury and suggests that further study incorporating a consideration of occupational, residential, and changing nutritional exposures is needed.

## 5. Conclusions

Low levels of total hair mercury in the control study population near the headwaters, where ASGM mining was not occurring during the study period, suggest that higher total hair mercury levels observed in communities were associated with the presence of ASGM mining. We did not observe the levels of total hair mercury to be related to levels of mercury in the local fish. Our inability to assess the ASGM status among individual subjects limits our ability to evaluate the impact of ASGM activities on individual doses of total hair mercury, and may have resulted in an underestimation of mercury exposure in the subjects of the three ASGM communities assessed. The frequency of the consumption of fish and the number of fish consumed did not prove to be predictive of observed levels of total mercury in hair. More studies are needed to determine what species of fish, and what sources of fish, are lowest in mercury concentration for human consumption.

In all 111 observations of farmed fish in the region of Madre de Dios, there were no levels of mercury above the EPA Tissue Residue Criterion, though it is important to note that the fish sampled were only, on average, about one-half the age of harvest in the region of Madre de Dios, and so our estimates of mercury levels in fish at the time of harvest for sale or consumption are likely to be low. Also, the fish ponds sampled as part of our study were relatively new (a few years old on average), and it is likely that, as the ponds age, methylmercury will continue to accumulate through transformation after atmospheric deposition in the stagnated pond water. Finally, paco are omnivorous fish, and so it is expected that they would have a lower mercury concentration under natural conditions than other carnivorous fish.

Our study has shown that there may be an increased risk of mercury exposure in some populations in the region of Madre de Dios; this risk appears to be dependent upon location. Further research on historical mercury levels in sediments throughout the river basin, localized biotic and abiotic factors affecting environmental chemical processes, and the analysis of similar populations along the main channel of the Madre de Dios River, as well as its tributaries, would help to definitively quantify the impact that anthropogenic releases of mercury from ASGM has on the human population and ecosystem.

## Figures and Tables

**Table 1:** Geographic and mining-related characteristics of four human study sites in the Madre de Dios river basin of Southeastern Peru.

Study Site	GPS Coordinates	Distance ^1^ to Headwaters (km)	District	Region	Illegal Mining Activity
Bajo Madre de Dios	12°35′639″ S 69°09′197″ W	228.1	Tambopata	Madre de Dios	Yes
Boca Amigo	12°36′161″ S 70°05′288″ W	142.8	Madre de Dios	Madre de Dios	Yes
Mazuco	13°06′558″ S 70°22′770″ W	69.4	Inambari	Madre de Dios	Yes
Pilcopata	12°54′367″ S 71°24′178″ W	53.4	Kosñipata	Cusco	No

^1^ Calculated in ArcGIS10 software using a Haversine formula to calculate the great-circle distance between the study site coordinates and those of the Madre de Dios river origin (ignores topography and river bends; “As the crow flies”). Although these numbers are not the actual distance downriver, they did correctly rank-order the villages (i.e., closest to furthest from the headwaters), which is consistent with the variable created in our analysis. The community of Mazuco is located on the Inambari River, a tributary to the Madre de Dios River. Similarly, the community of Pilcopata is located on the Kosñipata River, a tributary to the Upper Madre de Dios River, which flows into the main Madre de Dios River. Thus, the purpose of the “distance to headwaters” measurement is not intended to be a measurement of distance along the Madre de Dios River for a community, but rather to highlight the distance from the point of origin of the Madre de Dios River.

**Table 2:** Characteristics of 24 study sites for *Piaractus brachypomus* (Paco) sample collection in the Madre de Dios river basin of Southeastern Peru.

Site	No. of Farms	No. of Samples	GPS
Iberia	2	10	11°24′629″ S 69°23′026″ W
Puerto Maldonado	3	10	29°39′033″ S 69°19′290″ W
Mazuco	1	5	13°03′968″ S 70°21′145″ W
Nueva Generación	1	5	12°50′225″ S 70°11′766″ W
Primavera Alta	2	10	12°54′765″ S 70°09′002″ W
Primavera Baja	4	17	12°54′805″ S 70°10′285″ W
Santa Rosa	2	10	12°53′227″ S 70°18′669″ W
Santa Rita Alta	3	14	12°54′556″ S 70°14′529″ W
Santa Rita Baja	2	9	12°54′814″ S 70°12′360″ W
Virgen de la Candalaria	2	11	12°52′735″ S 70°01′987″ W
Villa Santiago	2	10	13°01′621″ S 70°20′954″ W
Total	24	111	

**Table 3:** Demographic characteristics of 81 human participants at four study sites in the Madre de Dios river basin of Southeastern Peru.

**Site**	**N**	**n Male (%)**	**Mean (SD) Age (Years)**	**Mean (SD) Residence (Years)**	**Mean (SD) n Fish Servings in Past 3 Days (SD) ^1^**	**Mean n (SD) in Household**	
						**Total**	**Children ^2^**
Overall	81	39 (48.2)	43.3 (17.7)	16.5 (16.9)	1.7 (2.1)	3.9 (2.5)	1.4 (1.5)
Bajo Madre de Dios	21	13 (61.9)	58.8 (16.3)	23.5 (18.9)	1.3 (2.2)	3.1 (2.2)	0.9 (1.3)
Boca Amigo	17	7 (41.2)	34.2 (10.7)	3.2 (2.5)	3.0 (2.7)	3.5 (2.0)	1.0 (0.9)
Mazuco	23	10 (43.5)	41.5 (18.0)	24.6 (19.0)	1.9 (2.0)	4.3 (2.6)	1.5 (1.5)
Pilcopata	20	6 (30.0)	36.6 (13.5)	11.1 (8.8)	0.9 (1.0)	4.6 (3.1)	2.1 (1.8)
**Site**	**N (%) Eating Fish Species**						
	**Paco**		**Bagre**	**Boca chico ^3^**	**Sabalo ^3^**	**Doncella ^3^**	**Zungaro ^3^**
	37 (45.7)		28 (34.6)	28 (34.6)	18 (22.2)	21 (25.9)	20 (24.7)
Overall	13 (61.9)		10 (47.6)	6 (28.6)	7 (33.3)	10 (47.6)	8 (38.1)
Bajo Madre de Dios	7 (41.2)		8 (47.1)	5 (29.4)	0 (0.0)	10 58.8)	9 (52.9)
Boca Amigo	7 (30.4)		3 (13.0)	17 (73.9)	10 (43.5)	0 (0.0)	1 (4.3)
Mazuco	10 (50.0)		7 (35.0)	0 (0.0)	1 (5.0)	1 (5.0)	2 (10)
Pilcopata							

^1^ Significant difference among sites, ANOVA, *p*-value < 0.05; ^2^ Significant difference among sites, ANOVA, *p*-value < 0.01; ^3^ Significant difference among sites, χ^2^, *p*-value < 0.01.

**Table 4:** Descriptive statistics for the mercury (µg Hg/g hair) in human hair samples by site and sex, at four study sites in the Madre de Dios river basin of Southeastern Peru.

Site/Sex	n	Range	Arithmetic Mean ± SD	Geometric Mean ± GSD	No. (%) ≥ Reference Level of 2.2 µg Hg/g Hair
Overall ^1^	80	0.3, 11.0	3.4 ± 3.0	2.0 ± 2.5	36 (45.0) ^3^
Male	39	0.3, 11.0	3.1 ± 2.8	2.0 ± 2.8	19 (48.7)
Female	41	0.5, 10.1	3.6 ± 3.4	2.1 ± 2.3	17 (41.5)
Bajo Madre de Dios	21	0.6, 11.0	4.3 ± 2.8	3.3 ± 2.2 ^2^	14 (66.7)
Male	13	0.6, 11.0	4.4 ± 0.6	3.2 ± 2.5	9 (69.2)
Female	8	1.3, 6.5	4.0 ± 0.5	3.5 ± 1.8	5 (62.5)
Boca Amigo ^1^	17	2.1, 10.1	5.0 ± 0.4	4.5 ± 1.6 ^2^	15 (93.8)
Male	7	2.3, 7.9	4.9 ± 0.5	4.6 ± 1.5	7 (100.0)
Female	10	2.1, 10.1	5.1 ± 0.6	4.5 ± 1.7	8 (88.9)
Pilcopata	23	0.3, 2.3	0.9 ± 0.5	0.8 ± 1.7 ^2^	1 (4.4)
Male	13	0.3, 2.3	0.9 ± 0.1	0.8 ± 0.6	1 (7.7)
Female	10	0.5, 1.6	1.0 ± 0.8	0.9 ± 1.5	0 (0.0)
Mazuco	20	0.6, 9.2	2.6 ± 2.3	1.9 ± 2.1 ^2^	6 (30.0)
Male	6	0.9, 9.2	3.2 ± 0.9	2.2 ± 2.3	2 (33.3)
Female	14	0.6, 7.3	2.3 ± 0.4	1.8 ± 2.1	4 (28.6)

^1^ Boca Amigo had one observation with an average total mercury content of 30.12 µg Hg/g hair. This outlier was not included in any of the above statistical calculations except for the toxic level count; ^2^ Significant difference in geometric mean total hair mercury levels between sites, ANOVA, *p* < 0.01; ^3^ Significant difference in fraction of samples ≥reference level between sites, χ^2^, *p* < 0.01.

**Table 5:** Results of multivariable linear and logistic regression models evaluating differences in total hair mercury at four study sites in the Madre de Dios river basin of Southeastern Peru.

**Model**	**Adjusted R^2^**	**Variables**	**Coefficient (SE)**	***p* Value**
Linear regression, dependent variable log-transformed total hair mercury				
1	0.50	Site (Reference: Pilcopata)		
		Bajo Madre de Dios	1.41 (0.22)	<0.0001
		Boca Amigo	1.73 (0.17)	<0.0001
		Mazuco	0.8 (0.20)	<0.0001
2	0.51	Site (Reference: Pilcopata)		
		Bajo Madre de Dios	1.45 (0.23)	<0.0001
		Boca Amigo	1.71 (0.18)	<0.0001
		Mazuco	0.84 (0.20)	<0.0001
		Age	−0.002 (0.005)	0.74
		Sex (reference: Male)	−0.004 (0.15)	0.98
**Model**	**Pseudo R^2^**	**Variables**	**Odds Ratio (SE)**	***p* Value**
Logistic regression, dependent variable total hair mercury level above reference level				
3	0.39	Site (Reference: Pilcopata)		
		Bajo Madre de Dios	3.78 (1.58–5.98)	0.001
		Boca Amigo	5.80 (2.95–8.64)	<0.0001
		Mazuco	2.24 (0.02–4.46)	0.04
4	0.40	Site (Reference: Pilcopata)		
		Bajo Madre de Dios	3.93 (1.58–6.28)	0.001
		Boca Amigo	5.91 (3.12–8.81)	<0.0001
		Mazuco	2.40 (0.13–4.67)	0.03
		Age	−0.007 (−0.05–0.04)	0.73
		Sex (reference: Male)	−0.64 (−5.33–0.23)	0.28

**Table 6:** Descriptive statistics for measured mercury concentration in paco fish tissue samples at 24 sites in the Madre de Dios river basin of Southeastern Peru.

Site	n	Range Ww [Hg] (mg/kg)	Arithmetic Mean ± SD Ww [Hg] (mg/kg)	Geometric Mean ± GSD Ww [Hg] (mg/kg) ^2^	No. (%) ≥ EPA Tissue Residue Criterion of 0.3 mg/kg
Overall	111	0.01, 0.23	0.05 ± 0.04	0.04 ± 1.3	0 (0)
Iberia	10	0.01, 0.09	0.04 ± 0.03	0.03 ± 1.5	0 (0)
Mazuco	5	0.02, 0.03	0.03 ± 0.01	0.03 ± 1.1	0 (0)
Puerto Maldonado ^1^	10	0.02, 0.10	0.04 ± 0.04	0.03 ± 1.4	0 (0)
Nueva Generación	5	0.05, 0.11	0.07 ± 0.02	0.07 ± 1.1	0 (0)
Primavera Alta	10	0.04, 0.12	0.07 ± 0.03	0.06 ± 1.2	0 (0)
Primavera Baja	18	0.03, 0.08	0.05 ± 0.02	0.04 ± 1.4	0 (0)
Santa Rosa	10	0.02, 0.05	0.03 ± 0.03	0.03 ± 1.2	0 (0)
Santa Rita Alta	14	0.02, 0.07	0.04 ± 0.01	0.04 ± 1.2	0 (0)
Santa Rita Baja	9	0.03, 0.09	0.04 ± 0.02	0.04 ± 1.2	0 (0)
Virgen de la Candalaria	11	0.05, 0.23	0.12 ± 0.06	0.10 ± 1.3	0 (0)
Villa Santiago	10	0.05, 0.06	0.05 ± 0.01	0.05 ± 1.0	0 (0)

^1^ One outlier was removed from the samples collected from this study site; 2 Significant difference in geometric mean levels by site, ANOVA, *p* < 0.001.

## Supplementary Materials

The following are available online at www.mdpi.com/1660-4601/14/3/302/s1, Figure S1: Comparison of mercury in paco fish tissue by study site: mean wet weight mercury concentration (mg/kg), mean dry weight mercury concentration (mg/kg).

## References

[1] Hentschel T., Hruschka F., Priester M. Global Report on Artisanal & Small-Scale Mining. http://pubs.iied.org/pdfs/G00723.pdf.

[2] United Nations Environment Programme (2012). Reducing Mercury Use in arTisanal and Small-Scale: A Practical Guide.

[3] International Labour Organization (1999). Report for Discussion at the Tripartite Meeting on Social and Labour Issues in Small-Scale Mines.

[4] Damonte G.H. (2016). Taming the “Wilderness”: Government Quest for Forrmalization and Conflict among Small-Scale Miners in the Peruvian Amazon. Antipode.

[5] United Nations Environment Programme (UNEP) (2012). Analysis of Formalization Approaches in the Artisanal and Small-Scale Gold Mining Sector Based on Experiences in Ecuador, Mongolia, Peru, Tanzania and Uganda: Peru Case Study.

[6] El Instituto Nacional de Estadística e Informática (INEI) (2010). PERÚ: Estimaciones y Proyecciones de Población Departamental, por Años Calendario y Edades Simples 1995–2025.

[7] Brooks W.E., Sandoval E., Yepez M.A., Howell H. (2007). Peru Mercury Inventory 2006.

[8] VERITE (2013). Risk Analysis of Indicators of Forced Labor and Human Trafficking in Illegal Gold Mining in Peru.

[9] Swenson J.J., Carter C.E., Domec J.C., Delgado C.I. (2011). Gold mining in the peruvian amazon: Global prices, deforestation, and mercury imports. PLoS ONE.

[10] Asner G.P., Llactayo W., Tupayachi R., Luna E.R. (2013). Elevated rates of gold mining in the Amazon revealed through high-resolution monitoring. Proc. Natl. Acad. Sci. USA.

[11] Veiga M.M., Maxson P.A., Hylander L.D. (2006). Origin and consumption of mercury in small-scale gold mining. J. Clean. Prod..

[12] Álvares J., Sotero V., Brack E.A., Ipenza P.C.A. (2011). Minería Aurífera en Madre de Dios y Contaminación con Mercurio.

[13] Olson D.M., Dinerstein E. (1998). The Global 200: A Representation Approach to Conserv. Conserv. Biol..

[14] Drake P.L., Rojas M., Reh C.M., Mueller C.A., Jenkins F.M. (2001). Occupational exposure to airborne mercury during gold mining operations near El Callao, Venezuela. Int. Arch. Occup. Environ. Health.

[15] Winfrey R., Rudd W.M. (1990). Formation of methylmercury in low pH lakes. Environ. Toxicol. Chem..

[16] Bloom N.S. (1992). On the Chemical Form of Mercury in Edible Fish and Marine Invertebrate Tissue. Can. J. Fish. Aquat. Sci..

[17] Pfeiffer W.C., Lacerda L.D. (1988). De Mercury inputs into the Amazon Region, Brazil. Environ. Technol. Lett..

[18] National Research Council Dose Estimation (2000). Toxicological Effects of Methylmercury.

[19] Pirrone N., Cinnirella S., Feng X., Finkelman R.B., Friedli H.R., Leaner J., Mason R., Mukherjee A.B., Stracher G.B., Streets D.G. (2010). Global mercury emissions to the atmosphere from anthropogenic and natural sources. Atmos. Chem. Phys..

[20] United Nations Environment Programme (UNEP) (2013). Global Mercury Assessment 2013: Sources, Emissions, Releases, and Environmental Transport.

[21] United Nations Environment Programme (UNEP) (2013). Minamata Convention on Mercury.

[22] Piñeiro V., Thomas J., Elverdin P. The Agricultural Sector as an Alternative to Illegal Mining in Peru: A Case Study of Madre de Dios. https://papers.ssrn.com/sol3/papers.cfm?abstract_id=2884337.

[23] Finer M., Olexy T., Novoa S. Gold Mining Deforestation within Tambopata National Reserve Exceeds 450 Hectares. http://maaproject.org/2016/tambopata450/.

[24] Fraser B. (2016). Peru’s gold rush raises health fears. Nature.

[25] Lebel J., Mergler D., Branches F., Lucotte M., Amorim M., Larribe F., Dolbec J. (1998). Neurotoxic effects of low-level methylmercury contamination in the Amazonian Basin. Environ. Res..

[26] Zahir F., Rizwi S.J., Haq S.K., Khan R.H. (2005). Low dose mercury toxicity and human health. Environ. Toxicol. Pharmacol..

[27] Salonen J.T., Seppanen K., Lakka T.A., Salonen R., Kaplan G.A. (2000). Mercury accumulation and accelerated progression of carotid atherosclerosis: A population-based prospective 4-year follow-up study in men in eastern Finland. Atherosclerosis.

[28] Oken E., Wright R.O., Kleinman K.P., Bellinger D., Amarasiriwardena C.J., Hu H., Rich-Edwards J.W., Gillman M.W. (2005). Maternal fish consumption, hair mercury, and infant cognition in a U.S. cohort. Environ. Health Perspect..

[29] Jarosińska D., Horvat M., Sällsten G., Mazzolai B., Dabkowska B., Prokopowicz A., Biesiada M., Barregård L. (2008). Urinary mercury and biomarkers of early renal dysfunction in environmentally and occupationally exposed adults: A three-country study. Environ. Res..

[30] Schwenk M., Klein R., Templeton D.M. (2009). Immunological effects of mercury (IUPAC Technical Report). Pure Appl. Chem..

[31] Basu N., Goodrich J.M., Head J. (2014). Ecogenetics of mercury: From genetic polymorphisms and epigenetics to risk assessment and decision-making. Environ. Toxicol. Chem..

[32] Liang Y., Sun R., Sun Y., Chen Z., Li L. (1993). Psychological effects of low exposure to mercury vapor: Application of a computer-administered neurobehavioral evaluation system. Environ. Res..

[33] Centers for Disease Control and Prevention Fourth National Report on Human Exposure to Environmental Chemicals. https://www.cdc.gov/biomonitoring/pdf/fourthreport_updatedtables_feb2015.pdf.

[34] Gilbert S.G., Grant-Webster K.S. (1995). Neurobehavioral effects of developmental methylmercury exposure. Environ. Health Perspect..

[35] Steuerwald U., Weihe P., Jørgensen P.J., Bjerve K., Brock J., Heinzow B., Budtz-Jørgensen E., Grandjean P. (2000). Maternal seafood diet, methylmercury exposure, and neonatal neurologic function. J. Pediatr..

[36] International Programme on Chemical Safety, WHO (1990). Methylmercury (Environmental Health Criteria 101).

[37] Caldwell K.L., Mortensen M.E., Jones R.L., Caudill S.P., Osterloh J.D. (2009). Total blood mercury concentrations in the U.S. population: 1999–2006. Int. J. Hyg. Environ. Health.

[38] Veiga M. Mercury in artisanal gold mining in Latin America: Facts, fantasies and solutions. Proceedings of the UNIDO—Expert Group Meeting—Introducing New Technologies for Abatement of Global Mercury Pollution Deriving from Artisanal Gold Mining.

[39] McDowell M.A., Dillon C.F., Osterloh J., Bolger P.M., Pellizzari E., Fernando R., Montes de Oca R., Schober S.E., Sinks T., Jones R.L. (2004). Hair mercury levels in U.S. children and women of childbearing age: Reference range data from NHANES 1999–2000. Environ. Health Perspect..

[40] World Health Organization (1990). Environmental Health Criteria, 101: Methylmercury.

[41] Joint FAO/WHO Expert Committee on Food Additives (2003). Sixty-First Meeting. Summary and Conclusions.

[42] Sheehan M.C., Burke T.A., Navas-Acien A., Breysse P.N., McGready J., Fox M.A. (2014). Global methylmercury exposure from seafood consumption and risk of developmental neurotoxicity: A systematic review. Bull. World Health Organ..

[43] Silva-Forsberg M.C., Forsberg B.R., Zeidemann V.K. (1999). Mercury Contamination in Humans Linked to River Chemistry in the Amazon Basin P. Ambio.

[44] Lechler P.J., Miller J.R., Lacerda L.D., Vinson D., Bonzongo J.C., Lyons W.B., Warwick J.J. (2000). Elevated mercury concentrations in soils, sediments, water, and fish of the Madeira River basin, Brazilian Amazon: A function of natural enrichments?. Sci. Total Environ..

[45] Centro de Atención Médica Especializada y Preventiva (2013). Mercury in Madre de Dios—Mercury Concentrations in Fish and Humans in Puerto Maldonado.

[46] Ashe K. (2012). Elevated mercury concentrations in humans of madre de dios, Peru. PLoS ONE.

[47] Yard E.E., Horton J., Schier J.G., Caldwell K., Sanchez C., Lewis L., Gastaňaga C. (2012). Mercury Exposure Among Artisanal Gold Miners in Madre de Dios, Peru: A Cross-sectional Study. J. Med. Toxicol..

[48] United States Environmental Protection Agency (2001). Water Quality Criterion for the Protection of Human Health: Methylmercury Final.

[49] Gutleb A.C., Schenck C., Staib E. (1997). Giant otter (Pteronura brasiliensis) at risk? Total mercury and methylmercury levels in fish and otter scats, Peru. Ambio.

[50] Luis Fernández V.G. Niveles del Mercurio en Peces de Madre de Dios. http://www.minam.gob.pe/mineriailegal/wp-content/uploads/sites/43/2013/10/Carnegie-mercurio-Madre-de-Dios.pdf.

[51] Roach K.A., Jacobsen N.F., Fiorello C.V., Stronza A., Winemiller K.O. (2013). Gold mining and mercury bioaccumulation in a floodplain lake and main channel of the Tambopata River, Perú. J. Environ. Prot..

[52] Kohler C.C., Kohler S.T., Alcantara F., Isern E.R. (1997). Development of sustainable pond aquaculture practices for Piaractus brachypomus in the Peruvian amazon. Pond Dynamics/Aquaculture Collaborative Research Support Program Fifthteenth Annual Technical Report.

[53] Diana J.S. (2009). Aquaculture Production and Biodiversity Conservation. Bioscience.

[54] African Cashew Alliance Annual Report 2012. http://www.africancashewalliance.com/sites/default/files/documents/aca-annual-report-2012.pdf.

[55] Flores H.G., Bocanegra F.A., Riveiro H.S., Quiroz S.A. (1992). Hibridacion de paco, Piaractus brachypomus (Cuvier, 1818) por gamitana, Colossoma macropomum (Cuvier, 1818) En Iquitos -Peru. Folia Amaz..

[56] Morel F.M.M., Kraepiel A.M.L., Amyot M. (1998). The Chemical Cycle and Bioaccumulation of Mercury. Annu. Rev. Ecol. Syst..

[57] Ziesler R., Ardizzone G.D. (1979). The Inland Waters of Latin America.

[58] AABP Atrium Biodiversity Information System for the Andes to Amazon Biodiversity Program at the Botanical Research Institute of Texas. http://www.atrium-biodiversity.org/.

[59] US Environmental Protection Agency (2007). Method 7473, mercury in solids and solutions by thermal decomposition, amalgamation, and atomic absorption spectometry. Test Methods for Evaluating Solid Waste, Physical/Chemical Methods.

[60] World Health Organization (2008). Guidance for Identifying Populations at Risk from Mercury Exposure.

[61] Sun G.W., Shook T.L., Kay G.L. (1996). Inappropriate use of bivariable analysis to screen risk factors for use in multivariable analysis. J. Clin. Epidemiol..

[62] Maldonado G., Greenland S. (1993). Simulation study of confounder-selection strategies. Am. J. Epidemiol..

