# Supplementary material for: Mercury Levels in Human Hair and Farmed Fish near Artisanal and Small-Scale Gold Mining Communities in the Madre de Dios River Basin, Peru
Source: Int J Environ Res Public Health. 2017 Mar 14;14(3):302. doi: 10.3390/ijerph14030302 (PMC5369138; doi:10.3390/ijerph14030302)
Supplement: Supplementary file 1 [file ijerph-14-00302-s001.docx]

**SUPPLEMENTAL INFORMATION**

**Figure S1:**  Comparison of mercury in paco fish tissue by study site: mean wet weight mercury concentration (mg/kg), mean dry weight mercury concentration (mg/kg)
